# MicroRNA-Dependent Mechanisms Underlying the Function of a β-Amino Carbonyl Compound in Glioblastoma Cells

**DOI:** 10.1021/acsomega.4c02991

**Published:** 2024-07-15

**Authors:** Denis Mustafov, Shoib S. Siddiqui, Andreas Kukol, George I. Lambrou, Shagufta, Irshad Ahmad, Maria Braoudaki

**Affiliations:** † School of Life and Medical Sciences, University of Hertfordshire, Hatfield, AL10 9AB, United Kingdom; ‡ College of Health, Medicine and Life Sciences, Brunel University London, Uxbridge UB8 3PH, United Kingdom; § Choremeio Research Laboratory, First Department of Pediatrics, School of Medicine, National and Kapodistrian University of Athens, Athens, Greece, Thivon and Levadeias 8, Goudi, 11527 Athens, Greece; ∥ University Research Institute of Maternal and Child Health and Precision Medicine, National and Kapodistrian University of Athens, Thivon and Levadeias 8, 11527 Athens, Greece; ⊥ Department of Biotechnology, School of Arts and Sciences, American University of Ras Al Khaimah, Ras Al Khaimah, United Arab Emirates

## Abstract

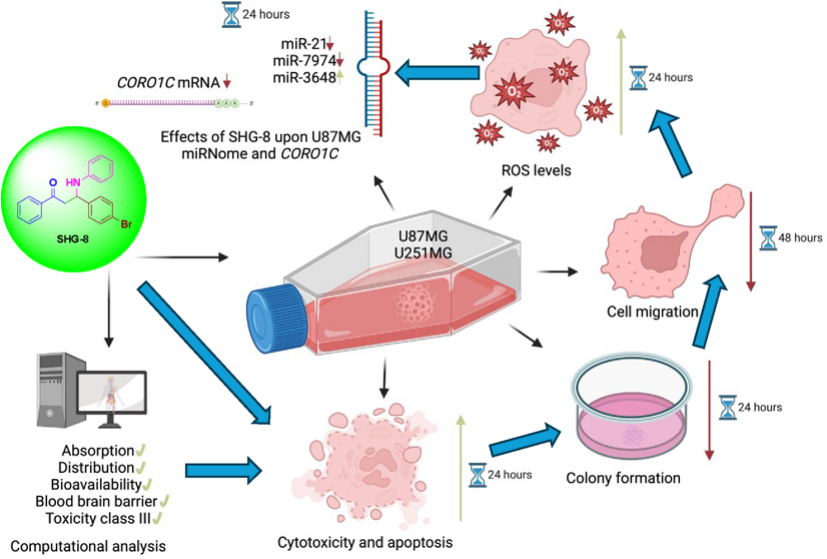

Glioblastoma (GB) is an aggressive brain malignancy characterized by its invasive nature. Current treatment has limited effectiveness, resulting in poor patients’ prognoses. β-Amino carbonyl (β-AC) compounds have gained attention due to their potential anticancerous properties. *In vitro* assays were performed to evaluate the effects of an in-house synthesized β-AC compound, named SHG-8, upon GB cells. Small RNA sequencing (sRNA-seq) and biocomputational analyses investigated the effects of SHG-8 upon the miRNome and its bioavailability within the human body. SHG-8 exhibited significant cytotoxicity and inhibition of cell migration and proliferation in U87MG and U251MG GB cells. GB cells treated with the compound released significant amounts of reactive oxygen species (ROS). Annexin V and acridine orange/ethidium bromide staining also demonstrated that the compound led to apoptosis. sRNA-seq revealed a shift in microRNA (miRNA) expression profiles upon SHG-8 treatment and significant upregulation of miR-3648 and downregulation of miR-7973. Real-time polymerase chain reaction (RT-qPCR) demonstrated a significant downregulation of *CORO1C*, an oncogene and a player in the Wnt/β-catenin pathway. *In silico* analysis indicated SHG-8’s potential to cross the blood–brain barrier. We concluded that SHG-8’s inhibitory effects on GB cells may involve the deregulation of various miRNAs and the inhibition of *CORO1C*.

## 1 Introduction

Glioblastoma (GB) is the most aggressive, fastest growing, and heterogeneous primary brain malignancy occurring in adults. GB accounts for nearly 3350 newly diagnosed cases in the UK annually with an overall survival rate between 6–17 months.^1^ Current, aggressive therapeutic approaches involving maximal surgical excision followed by radiotherapy and chemotherapy with temozolomide (TMZ) have failed to improve the overall survival rates, and patients’ prognoses remain poor.^2^ Furthermore, cisplatin is a highly effective chemotherapy drug. However, its application in treating GB is constrained by significant systemic toxicity and a limited ability to penetrate the brain tumor tissue, even with direct administration into the brain using standard delivery methods.^3^ Thus, the essential need for researching alternative therapeutic strategies for managing the malignancy have brought attention to microRNAs (miRNAs) due to their multimodal targeting capabilities.

MiRNAs are short, noncoding RNAs that regulate various biological mechanisms at the post-transcriptional level.^4^ In GB, the miRNA landscape reflects the disease stage and holds potential for prognostic evaluation and therapy selection. Previous research has demonstrated that miRNAs undergo dynamic alterations throughout the progression of the malignancy and have a direct involvement in GB tumorigenesis via regulating neo-angiogenesis, proliferation, invasion, and apoptosis.^5^

The functional relevance of GB-specific miRNAs highlights their dual roles as oncogenes and tumor-suppressors. Subsequently, miRNA expression profiling in GB aims to identify particular miRNA expression signatures associated with the tumorigenesis process and response to treatment. Several miRNAs have been found deregulated in GB and were associated with anticancer therapies. For instance, inhibition of miR-21 has been shown to increase the chemosensitivity of glioma cells to carmustine (BCNU), thereby enhancing the effectiveness of treatment.^6^ Another example to sensitize GB cells toward TMZ chemotherapy is the overexpression of miR-128.^7^ Thus, changes in the expression of miRNAs could underline their crucial role as therapeutic targets in GB and their potential in improving patient outcomes.

Meanwhile, the effectiveness of small-molecule inhibitors on miRNA expression remains a challenge and provides alternative therapeutic avenues. The biologically and medicinally important β-amino carbonyl (β-AC) compounds possess a β-amino carbonyl motif, which provides considerable stability and rigidity to the compound, resulting in enhanced pharmacokinetic dynamics and improved bioavailability.^8^ Current research has demonstrated that β-AC compounds can act as anticancer agents by interfering with mitochondrial structures, thereby exhibiting antineoplastic properties. Specifically, some of these molecules decrease the mitochondrial membrane potential and generate reactive oxygen species (ROS), leading to programmed cell death.^9^ The outcome of this cellular carbonyl stress might alter miRNA expression patterns and thus lead to their up- or downregulation. Further, oxidative stress might influence the miRNAs’ stability and consequently their interactions with their mRNA targets.^10^ To the best of our knowledge, the effects of β-AC compounds on miRNA regulation within cancer and particularly GB have not been studied before.

Several studies have reported the effectiveness of compounds possessing a carbonyl motif in GB management. For instance, the α,β-unsaturated carbonyl moiety of guaianolide sesquiterpene lactone cynaropicrin (1) expressed strong antiproliferative activity within U251MG glioblastoma cancer stem cells, suggesting potential therapeutic applications in targeting GB.^11^ In addition, a different sesquiterpene compound, compound 12 (oxyphyllanene B, OLB), which also possess an α,β-unsaturated carbonyl moiety was observed to induce apoptosis in time- and dose-dependent manners within TMZ-resistant GB cells.^12^

In the current study, we sought to investigate the efficacy of an in-house synthesized β-AC compound, b3-(4-bromophenyl)-1-phenyl-3-(phenylamino)propan-1-one (SHG-8), and its effects upon miRNA regulation within GB cell models. The results demonstrated reduced cell migration and proliferation alongside induced cell death via ROS. The miRNA expression levels were drastically influenced by the drug with miR-21 being significantly downregulated after exposure to SHG-8.

## 2 Materials and Methods

### 2.1 Synthesis of SHG-8

Considering the United Nations Sustainable Development Goals (SDGs), we have adopted a solid acid catalytic approach instead of Brønsted acid to synthesize SHG-8 (Supplementary Figure 1). The desired compound SHG-8 was synthesized by utilizing the sustainable sulfonic-acid-functionalized silica nanospheres (SAFSNS) nanocatalyst, which was prepared and characterized by Ahmad et al.^13^ Details on the synthesis of SHG-8 can be found in the Supplementary Methodology (SM1).

### 2.2 Cell Culture

The U87MG and U251MG glioblastoma cell lines isolated from malignant gliomas were derived from the American Type Culture Collection (ATCC, Manassas, VA, USA). The cells were cultured in complete minimum essential medium (MEM; GibcoTM, Bleiswijk, NL) supplemented with 10% fetal bovine serum (FBS) (Gibco, Bleiswijk, Netherlands) and 1% penicillin/streptomycin (Gibco, Bleiswijk, Netherlands). Throughout the study, the cells were kept in a humidifying incubator at 37 °C with 5% CO_2_ and were confirmed to be free of mycoplasma contamination.

### 2.3 *In Vitro* Functional Assays

Cell viability, colony forming, and wound-healing assays were performed as previously described by Vazhappilly et al.^14^ U87MG and U251MG GB cells were seeded at different cell densities and subsequently treated with increasing SHG-8 concentrations (diluted in DMSO, Dubai, UAE). Dimethyl sulfoxide (DMSO; Thermofisher, CA, USA) was used as a negative control, whereas cisplatin (200 μM; Sigma-Aldrich, Dorset, UK) was used as a positive control. Further details on how the functional assays were performed can be found in the Supplementary Methodology (SM2–SM4).

### 2.4 Determination of ROS

A ROS assay kit (Abcam, Cambridge, UK) was used to assess the release of ROS within glioblastoma cell lines. U87MG and U251MG cells (1.5 × 10^4^/well) were seeded in 96-well plates and incubated with 20 μL of Red ROS dye diluted in DMSO according to manufacturers’ guidance. The plates were incubated for 1 h at 5% CO_2_ and 37 °C. Subsequently, cells were treated with increasing SHG-8 concentrations (20 μM, 40 μM, 60 μM, 80 μM, and 100 μM), alongside PBS (negative control) and 1 mM H_2_O_2_ (positive control, Sigma-AldrichTM, Dorset, UK) for 30 min. Fluorescence increase of ROS was detected at an excitation wavelength of 520 nm and an emission wavelength of 605 nm using a CLARIOstar microplate reader at 0, 15, 30, 45, 60, 75, 90, 105, and 120 min.

### 2.5 Acridine Orange/Ethidium Bromide Staining

U87MG and U251MG cells were seeded at a density of 5 × 10^5^ cells/well in six-well chambers with DMSO (negative control), 200 μM cisplatin (positive control), and SHG-8 (50 μM and 100 μM) for 24 h. Post-treatment, the media were removed, and the cells were washed twice with PBS. Following washes, cells were trypsinized and pelleted. The cells were centrifuged at 200*g* for 5 min. The pellets were then resuspended in 1 mL of ice-cold PBS and centrifuged again at 200*g* for 5 min. The pellets were then resuspended in 20 μL of ice-cold PBS and stained with 2 μL of acridine orange (100 μg/mL) and ethidium bromide (100 μg/mL; ThermoFisher, CA, USA) dye for 3 min at room temperature. The stained cell suspension was added on a glass slide. The coverslips were applied onto the slides, and immunofluorescence images were captured using a 40× objective on an EVOS fluorescent microscope (Life Technologies, CA, USA) that was set at 30 lx/s exposure.

### 2.6 Annexin-V/Propidium Iodide Apoptosis Assay

To assess the extent of apoptosis induction following SHG-8 treatment, U87MG and U251MG cells were seeded at a density of 3 × 10^6^ cells/well in six-well chambers and allowed to adhere overnight. Subsequently, the wells containing cells were subjected to treatments with DMSO (negative control), 200 μM cisplatin (positive control), and SHG-8 (50 μM and 100 μM) for 24 h. Post-treatment, cells were washed twice with ice cold PBS, and 1 × 10^6^ cells were harvested in 100 μL of suspension. RNase (1 μL, 10 mg/mL) was applied to each cell suspension, followed by labeling with Annexin-V FITC (5 μL) and propidium iodide (100 μg/mL). As per manufacturer instructions, each cell suspension was incubated with the dead cells Annexin-V/propidium iodide apoptosis kit for flow cytometry (Invitrogen, Inchinnan, UK) at room temperature for 15 min. After the incubation period, 400 μL of annexin binding buffer was applied to each sample and subsequently analyzed using the Guavasoft 3.1.1 software and the guava easyCyte HT system (Merck Millipore, Watford, UK).

### 2.7 RNA Isolation and TaqMan Expression Assays

RT-qPCR was performed as described by Braoudaki et al.^15^ In brief, total RNA and miRNAs were extracted following the Trizol reagent (Ambion Life Technology, Aukland, New Zealand) protocol and mirVana isolation kit (ThermoFisher, Vilnius, Lithuania), respectively. The sample’s quantity and quality were assessed using Nanodrop (Nanodrop ND1000 Spectrophotometer, Marshall Scientific, Hampton, USA). Further details on RNA isolation and expression analysis can be found in the Supplementary Methodology (SM5).

### 2.8 Preparation of Samples for Small RNA Sequencing

U87MG RNA samples were extracted with Trizol. A nanodrop bioanalyzer was used to detect the purity of RNA samples, concentration, and integrity and to ensure the use of qualified samples for sequencing. RNA integrity was assessed using the RNA Screen Tape Kit of the Agilent Bioanalyzer 2100 system (Agilent Technologies, CA, USA). Samples with RNA integrity numbers (RINs) greater than seven were sent off for sRNA-seq to Biomarker Technologies (Biomarker Technologies, Germany). Further information on library preparation, clustering, sequencing, and data analyses can be found in the Supporting Information (SM6–13).

### 2.9 Metabolite and Toxicity Prediction of SHG-8

Metabolites of SHG-8 were predicted with Biotransformer 3.1.0 using the canonical Simplified Molecular-Input Line-Entry System (SMILES).^16^ The set of “Human and Human Gut” metabolic transformations was selected with one reaction iteration and a “combined CYP450 mode”. The absorption, distribution, metabolism, excretion, and toxicity (ADMET) properties of SHG-8 and metabolites were predicted with admetSAR 2.0 using the canonical SMILES with the option “ADMET properties for drug discovery” selected. AdmetSAR 2.0 predictions are based on machine learning methods based on molecular similarity utilizing data for 96 000 compounds.^17^

### 2.10 Molecular Docking Analysis

The crystal structure of human CORO1C was retrieved from the Protein Data Bank (https://www.rcsb.org). Docking analysis was then performed via utilizing CB-DOCK2 (https://cadd.labshare.cn/cb-dock2/index.php) to predict the stable binding configuration and molecular interactions between the receptor (CORO1C) and ligand (SHG-8). An SDF file of the query ligand was uploaded on CB-DOCK2, and blind docking was performed. The outcomes of the molecular docking between SHG-8 and the target protein were depicted using BioLip (version of 2021.09.15).^18^

### 2.11 Statistical Analysis

Statistical analysis was performed using GraphPad Prism 9.4.1 software (GraphPad Software, San Diego, USA), which was also used to create all graphical representations. One-way ANOVA was used to determine statistical differences between two groups, while two-way ANOVA tests followed by Tukey’s multiple comparisons were used for the analysis of three or more groups; *p* values of <0.05 were regarded as statistically significant.

## 3 Results and Discussion

### 3.1 SHG-8 Cytotoxicity Inhibited U251MG and U87MG Cells’ Proliferative Ability

To investigate whether SHG-8 exhibited cytotoxic effects within GB cells, an MTT cell viability assay was performed in the U251MG and U87MG cell lines treated with DMSO (as a vehicle, which corresponds to 0 μM SHG-8) and increasing SHG-8 concentrations. In particular, for the U251MG cell line, the concentrations tested were 50 μM, 100 μM, 150 μM, 200 μM, 250 μM, and 300 μM. Respectively, for the U87MG cell line, the concentrations tested were 20 μM, 40 μM, 80 μM, 100 μM, 120 μM, and 140 μM. In addition, both cell lines were treated with cisplatin and, in particular, at 50 μM, 100 μM, 150 μM, 200 μM, 250 μM, and 300 μM for both cell lines.

**Figure 1:**
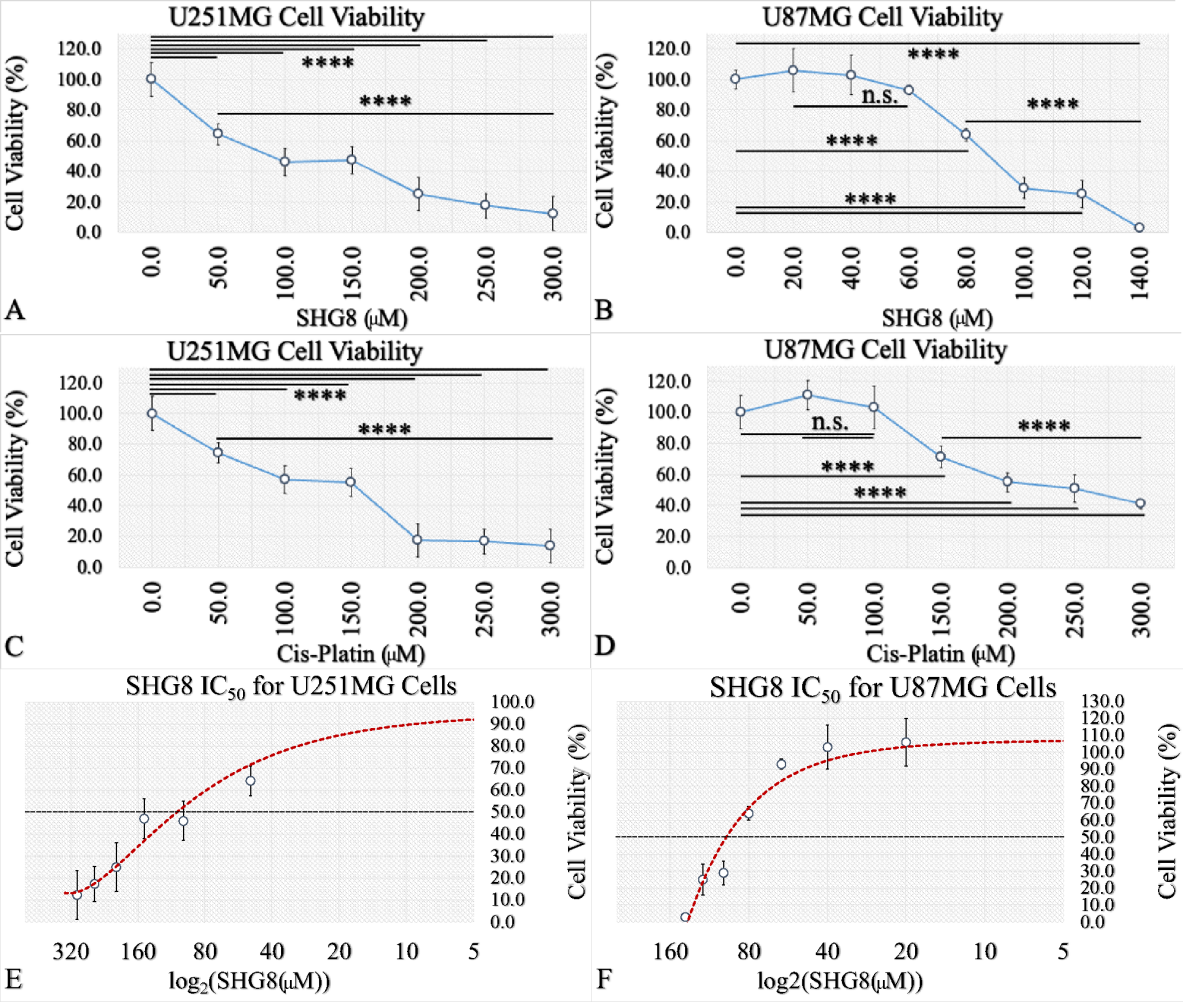
U251MG and U87MG glioma cell viability and proliferation dependency on SHG-8 concentration. MTT assay upon treatment with different concentrations of SHG-8 (50 μM, 100 μM, 150 μM, 200 μM, 250 μM, and 300 μM) for the U251MG cells revealing an IC_50_ of 100 μM (A, E). MTT assay upon treatment with SHG-8 (20 μM, 40 μM, 60 μM, 80 μM, 100 μM, 120 μM, and 140 μM) for the U87MG cells revealing an IC_50_ of 85 μM (B, F). MTT assay upon treatment with increasing concentrations of cisplatin (50 μM, 100 μM, 150 μM, 200 μM, 250 μM, and 300 μM) for both U251MG (C) as well as U87MG cells (D) manifested a significant cytotoxicity effect (legend: ns, nonsignificant, *p < 0.05, **p < 0.01, ***p < 0.001, ****p < 0.0001).

Cells treated with SHG-8, as obtained from the MTT assay, revealed a significant reduction of cell viability in the treatment conditions from 50 μM to 300 μM for U251MG cells (Figure 1A), as well as in the treatment conditions of 80 μM and 100 μM for the U87MG cell (Figure 1B). At the same time, U251MG cells appeared to be also sensitive to cisplatin (Figure 1C), with less sensitivity manifested by the U87MG cells (Figure 1D). For the U251MG cells, the IC_50_ of SHG-8 was estimated to be at the 100 μM level (*p* < 0.0001; Figure 1E), while the IC_50_ for U87MG cells was 85 μM (*p* < 0.0001; Figure 1F). Overall, both SHG-8 and cisplatin manifested significant cytotoxicity in both cell lines, yet the SHG-8 agent manifested better results in the U87MG cell line (Figure 1B).

### 3.2 SHG-8 Cytotoxicity Inhibits U251MG and U87MG Cells Colony Formation Abilities

**Figure 2:**
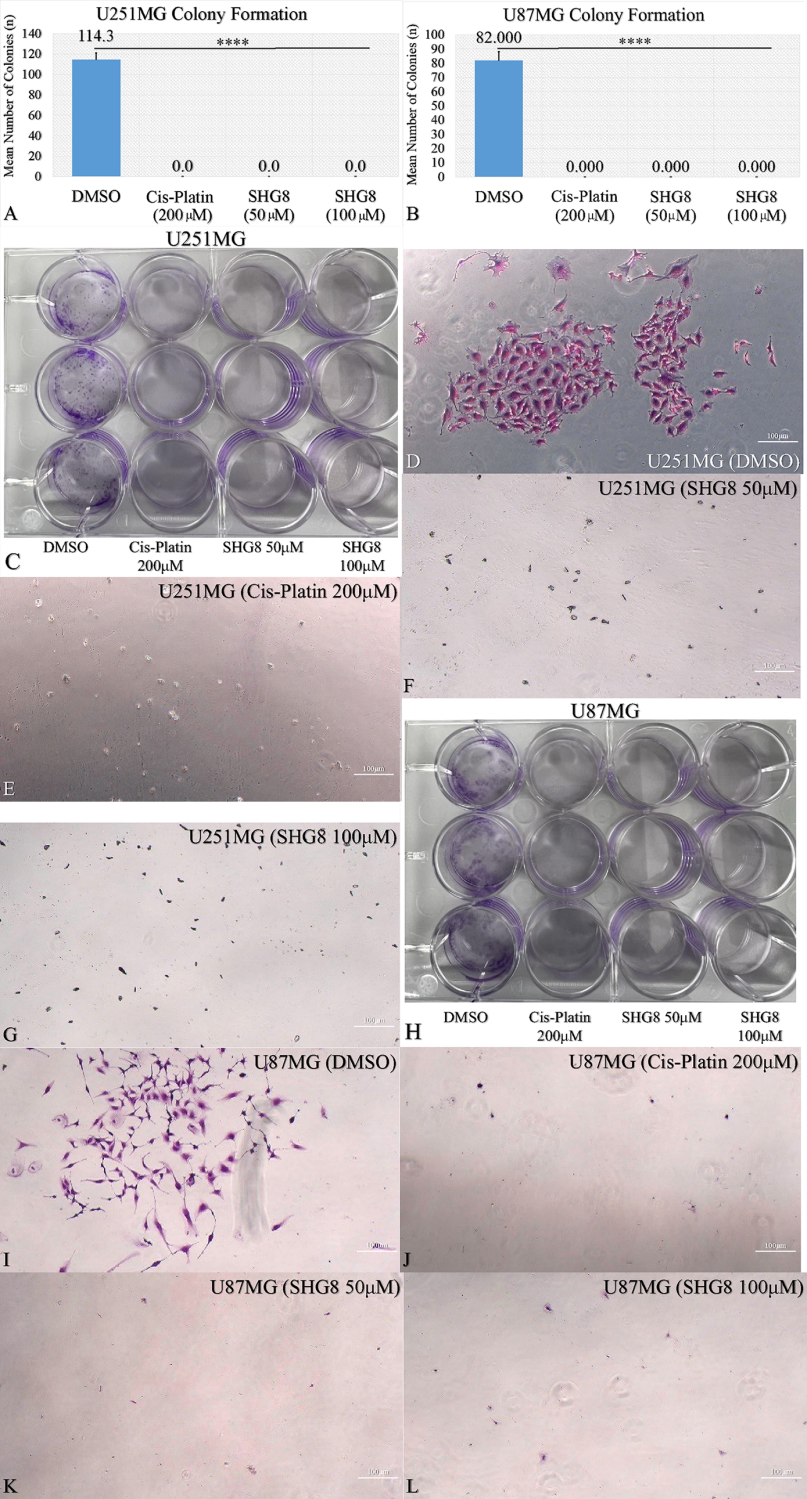
U251MG and U87MG glioma cell migration dependency on SHG-8 concentration. Significant decrease of U251MG colonies’ proliferative clonal capacity upon 50 μM and 100 μM SHG-8 treatment (A). Significant decrease of U87MG colonies’ proliferative clonal capacity upon 50 μM and 100 μM SHG-8 treatment (B). This was also manifested microscopically where the colony formation inhibition was observed for both the U251MG (C–G) and U87MG cells (H–L). Plates and microscopic images of colonies were taken under 40× magnification; legend: ns, nonsignificant, *p < 0.05, **p < 0.01, ***p < 0.001, ****p < 0.0001).

Following the observed cytotoxicity drug effects, the clonogenic capacity of U251MG and U871MG cells was examined after being treated with SHG-8 concentrations of 50 μM and 100 μM (Figure 2A–L). Cisplatin also significantly reduced the clonogenic abilities of GB cells (*p* < 0.0001).

### 3.3 SHG-8 Cytotoxicity Inhibits U251MG and U287 Cells Wound-Healing Capacity

**Figure 3:**
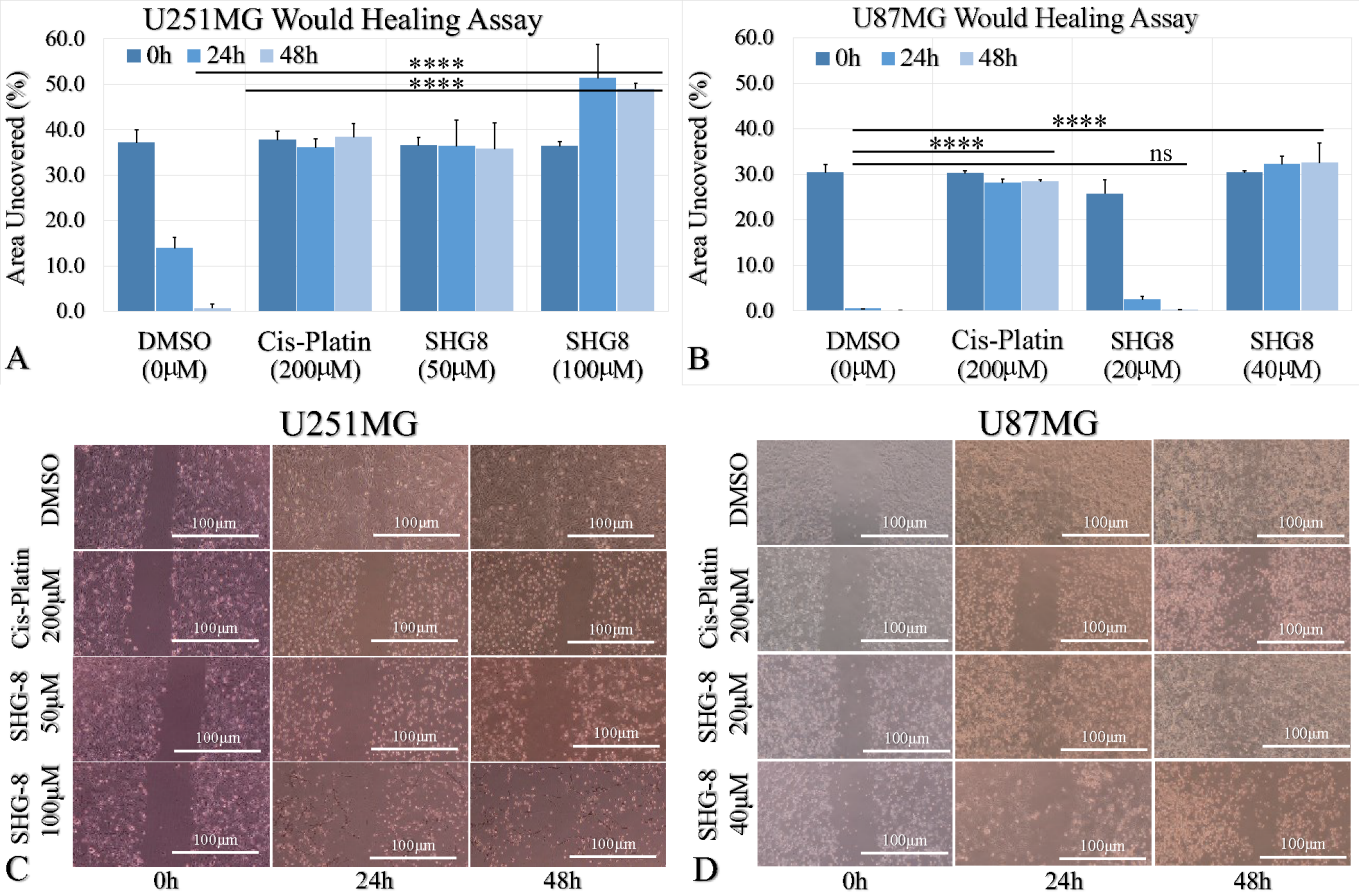
U251MG and U87MG glioma cells wound-healing assay dependency on SHG-8 concentration. (A) Quantification of the wound-healing assay of U251MG cells following treatment with DMSO, cisplatin, and SHG-8 (50 μM and 100 μM) across different time points. (B) Wound-healing area quantification of U87MG cells following treatment with DMSO, cisplatin, and SHG-8 (20 μM and 40 μM) across different time points. (C) Scratch images acquired at 0 h, 24 h and 48 h from U251MG cells treated as described above. (D) Wound (scratch) images acquired at 0 h, 24 h, and 48 h from U251MG cells treated as described above (legend: ns, nonsignificant; *p < 0.05, **p < 0.01, ***p < 0.001, ****p < 0.0001).

The wound-healing assay results demonstrated that the percentage of the area uncovered remained unchanged at 50 μM and 100 μM SHG-8 exposure (U251MG cells) and 40 μM SHG-8 exposure (U87MG cells) when compared to DMSO (Figure 3A and B). The results obtained post-treatment of U251MG cells revealed that concentrations of 50 μM and 100 μM both significantly seized cell migration and led to no change in the percentage of area uncovered post-48 h incubation when compared to DMSO control cells (*p* < 0.0001). In fact, there was apparent cell death at 100 μM SHG-8 concentration (Figure 3C). At 24 h, the relative percentage of area uncovered was less within control cells in comparison to U87MG cells treated with 20 μM and 40 μM SHG-8, respectively (*p* < 0.0001). At 48 h, the relative area uncovered of control cells decreased further; however, so did the scratch at 20 μM, whereas the gap at 40 μM remained unchanged (*p* < 0.0001), indicating a concentration-dependent inhibitory effect upon U87MG cell migration (Figure 3D).

Beta-amino carbonyl derivatives have demonstrated promising potential in medicinal chemistry, drug development, and cancer therapy owing to their diverse biological activities and capacity to trigger apoptosis in cancerous cells.^19,20^ This research incorporated an eco-friendly approach in an efficient, simple, and green catalytic process to produce SHG-8, aiming to demonstrate its *in vitro* cytotoxic effects on GB cell models.

The evaluation of the metabolic effects of SHG-8 upon GB cells revealed a significant decrease in cell viability and proliferation, suggesting that the β-AC compound possessed cytotoxic properties, which limit the growth and spread of cancer cells. Alongside the cytotoxic properties of the drug, the clonogenic potential of U87MG and U251MG cells was also significantly reduced, indicating impaired immortalization and proliferation of cancer cells. The dose-dependent decrease in cell viability indicated that higher concentrations of the SHG-8 compound were required to increase cell death. Similar cytotoxic effects were previously observed with several β-AC compounds on human prostate cancer cells and colorectal cancer cells.^20,21^ Currently, the most effective drug against gliomas is the chemotherapeutic agent TMZ. Combining chemotherapy with TMZ and radiotherapy is considered a gold standard therapeutic approach incorporated in the treatment regimens of GB patients with surgical tumor excision.^22^ Nasir et al. demonstrated that TMZ doses as high as 1000 μM were most effective in inhibiting cell viability in U87MG cells.^23^ The use of significantly high drug doses could lead to adverse effects on normal cells resulting in systemic toxicity, alongside intrinsic resistance to cytotoxic therapies. Subsequently, this contributes to treatment failure and tumor recurrence. Even though the actions of SHG-8 were observed to be dose-dependent, we demonstrated that concentrations below 100 μM were sufficient to induce restricted cell viability and cell proliferation due to induced cell cytotoxicity. These findings suggested SHG-8 can act effectively against brain tumor cells at low concentrations.

### 3.4 SHG-8 Induced Release of ROS and Led to Cell Death via Apoptosis

#### 3.4.1 ROS Release

**Figure 4:**
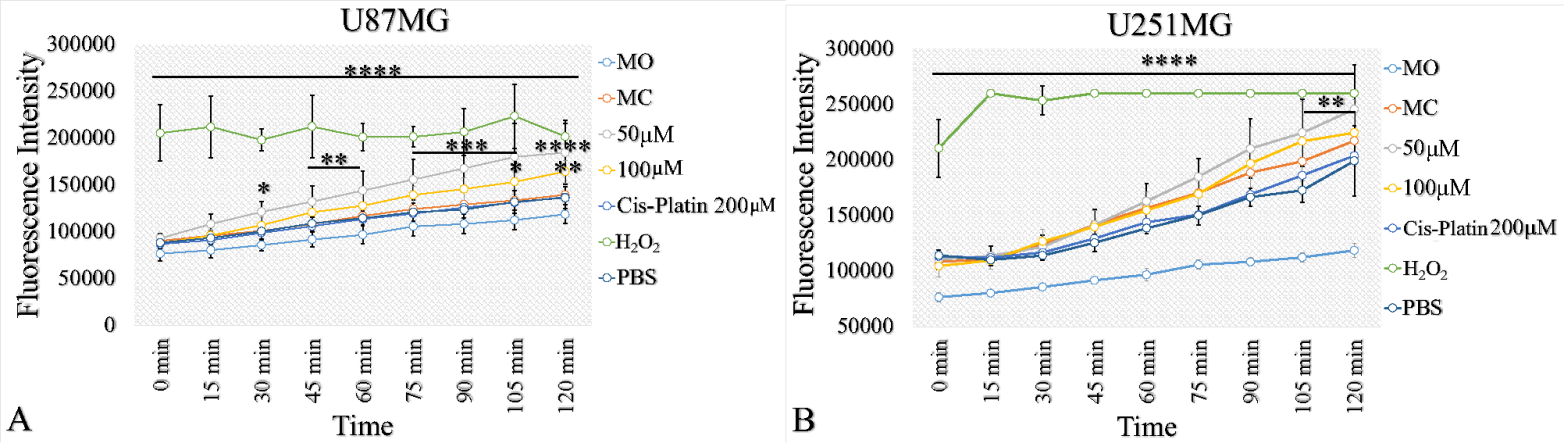
SHG-8 led to the release of ROS. (A) A significant but non-concentration-dependent elevation of ROS was observed post 30 min treatment period with 50 μM SHG-8 in comparison to the control condition. SHG-8 of 100 μM concentration induced ROS cytotoxicity at 90 min. At 120 min, both SHG-8 concentrations of 50 μM and 100 μM demonstrated significantly higher levels of ROS within U87MG. (B) A significant but non-concentration-dependent elevation of ROS observed post 105 min treatment period with 50 μM SHG-8 in comparison to the control condition for U251MG cells (legend: ns, nonsignificant; *p < 0.05, **p < 0.01, ***p < 0.001, ****p < 0.0001).

A significant elevation of ROS was observed during post 30 min treatment period of U87MG cells with 50 μM SHG-8 as compared to the control condition (Figure 4A). SHG-8 of 100 μM concentration induced ROS cytotoxicity later in time at 90 min. At 120 min, both SHG-8 concentrations demonstrated significantly higher levels of ROS within U87MG, resulting in oxidative damage of the U87MG GB cells. ROS release mechanisms of SHG-8-induced cytotoxicity in GB cells were observed to be time-dependent. A significant elevation for ROS was seen post 105 min within U251 treated cells with SHG-8 concentration at 50 μM (Figure 4B). Concentrations of 100 μM did not reveal significant ROS elevation for the later cell line.

#### 3.4.2 ROS-Related Apoptosis

**Figure 5:**
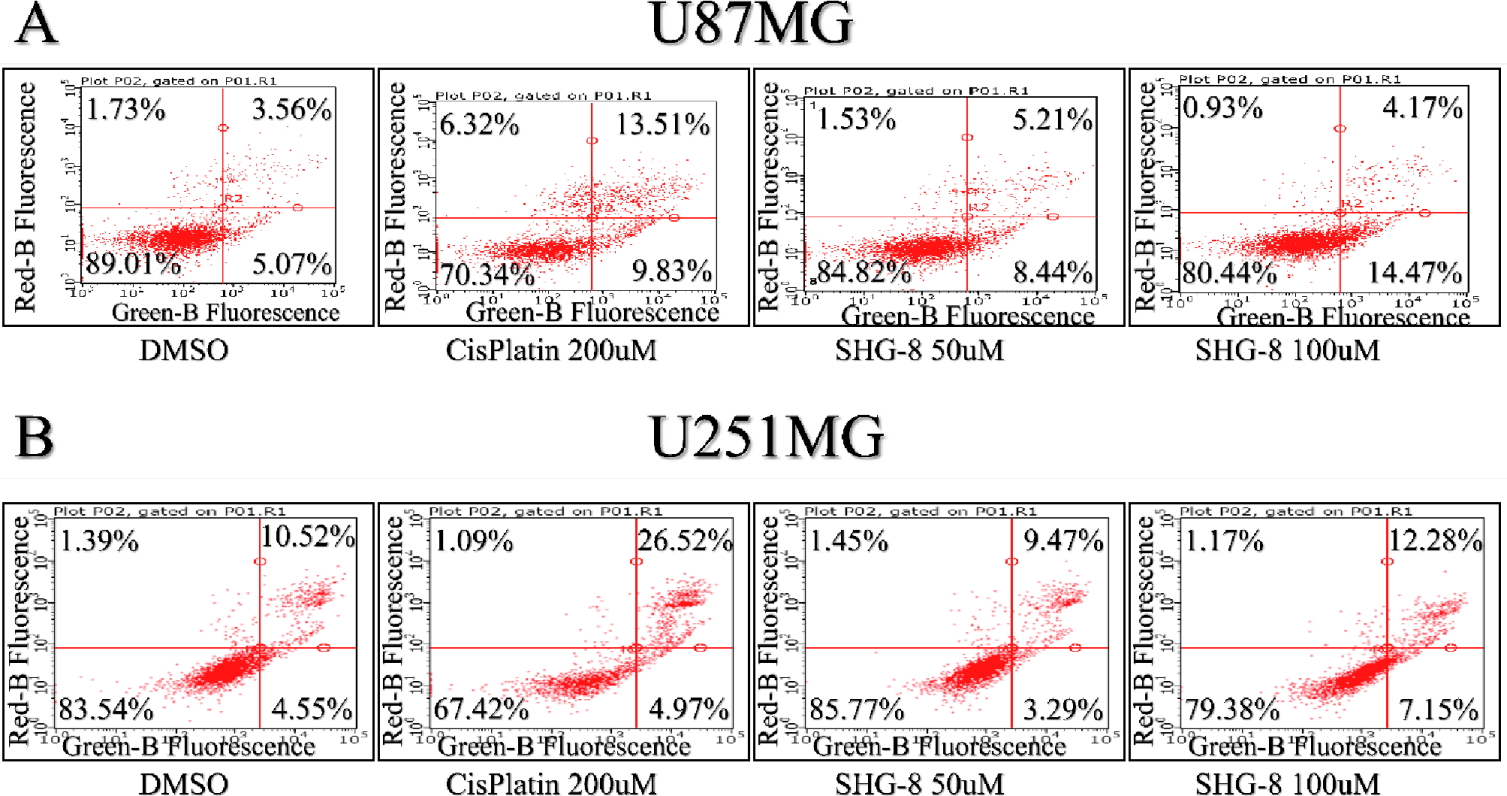
Annexin-V/propidium iodide (PI) staining of U87MG and U251MG cells treated with SHG-8. (A) Concentrations of 100 μM SHG-8 depicted a higher percentage of U87MG cells entering early apoptosis post-treatment (FITC positive and PI negative, lower right quadrant) in comparison to cisplatin, where more cells were seen in their late apoptosis stage (FITC positive and PI positive (upper right quadrant)). (B) Concentrations of 100 μM SHG-8 depicted a higher percentage of U251MG cells entering late apoptosis post-treatment (FITC positive and PI positive, upper right quadrant) in line with the positive control condition of cisplatin.

The Annexin V/PI assay revealed that U87MG cells treated with SHG-8 concentrations of 50 μM and 100 μM led to more cells entering an early apoptosis stage (8.44% and 14.47%, respectively; Figure 5A). The cisplatin positive control condition had a larger percentage of cells within the late apoptosis stage (13.51%). The degree of necrosis across all conditions was minimal. Cell cycle arrest analysis of U87MG cells demonstrated increased presence in the G2/M phase with SHG-8 at 50 μM. The results obtained from SHG-8-treated U251MG cells showed that more cells were entering late apoptosis with increasing SHG-8 concertation, similar to the results obtained from the positive control condition of cisplatin (Figure 5B). Cell cycle arrest was seen in the G2/M phase at 50 μM and 100 μM SHG-8-treated cells. It is notable that minor differences of the effect of SHG-8 on apoptosis between the two cell lines have been obtained, which could be attributed to variations in metabolic pathways such as glycolysis and purine metabolism. However, it is still evident that apoptosis was consistently detected in both cell lines, which validates the robustness of our results.

Compromising genomic integrity is a well-established cytotoxic mechanism of numerous genotoxic therapies.^24^ High levels of ROS have been previously detected in multiple neurodegenerative disorders, such as Alzheimer’s and Parkinson’s diseases.^25^ Thus, the therapeutic potential of ROS as a possible GB hallmark was evaluated in our study. To investigate if SHG-8 acted as a ROS-generating compound and its potential cytotoxic mechanism on DNA damage induction, ROS levels were measured upon treatment with the drug. Concentrations of 50 μM and 100 μM showed a significant impact on ROS release levels in a time-dependent manner. However, it is notable that 100 μM concentration induced ROS slower than did the 50 μM one, which could be attributed to the induced cell death caused by the higher concentration, resulting in less production of ROS. The antioxidant properties of ROS have also been observed in other inflammatory conditions, such as bowel disease.^26^ This is in line with this study’s findings, as GB is primarily associated with an inflammatory microenvironment signature that accelerates epigenetic changes, aiding tumor cells in avoiding immunological surveillance.^27^ The oxidative stress exhibited upon U87MG and U251MG cells via ROS in our treatment conditions was further supported via subsequent Annexing V/PI apoptosis assay. The SHG-8 treatment conditions (50 μM and 100 μM) resulted in the migration of U87MG cells toward the early apoptosis stage, while U251MG cells were seen to enter late apoptosis. These findings provide a potential explanation regarding the predominant cytotoxic mechanism of SHG-8 upon the inhibition of cell migration and proliferation, alongside the observed induction of apoptosis and tumor cell death.

### 3.5 Acridine Orange/EtBr (Ethidium Bromide) Staining

**Figure 6:**
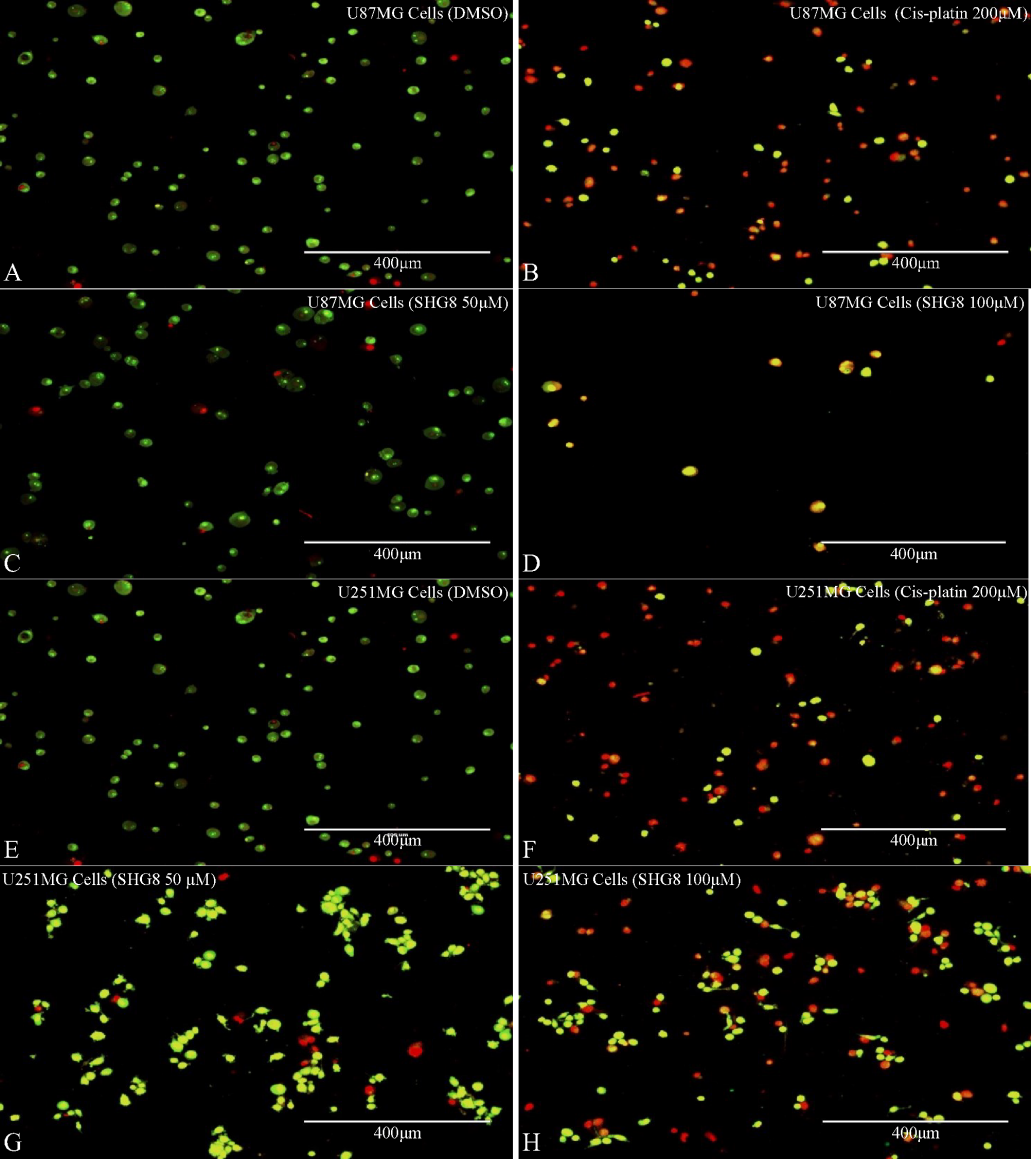
Acridine orange/ethidium bromide (AO/EB) staining. AO/EB fluorescent staining showing fluorescent microscopic images of U87MG and U251MG GB cells treated with DMSO (negative control), 200 μM cisplatin, and 50 μM and 100 μM of SHG-8 (A–H). Green cells were viable. Yellow cells indicated apoptosis, and orange/red cells indicated necrosis.

To detect if SHG-8 induced morphological alterations leading to apoptosis, AO/EtBr staining was performed for SHG-8-treated U87MG and U251MG cells (Figure 6A–H). The observed results depicted a high uptake of ethidium bromide at 100 μM SHG-8 concentrations, indicating a loss of membrane integrity and cell death. Concentrations of 50 μM depicted more U251MG cells entering early apoptosis in comparison to treated U87MG cells. Apoptotic and necrotic stages were also observed at 200 μM cisplatin, where more uptake of ethidium bromide was seen within U251MG treated cells. There was no indication of cell death in the negative control condition (DMSO), where the cells looked intact and in regular shape (Supplementary Table 2).

### 3.6 SHG-8 Downregulated miR-21 and *CORO1C*

**Figure 7:**
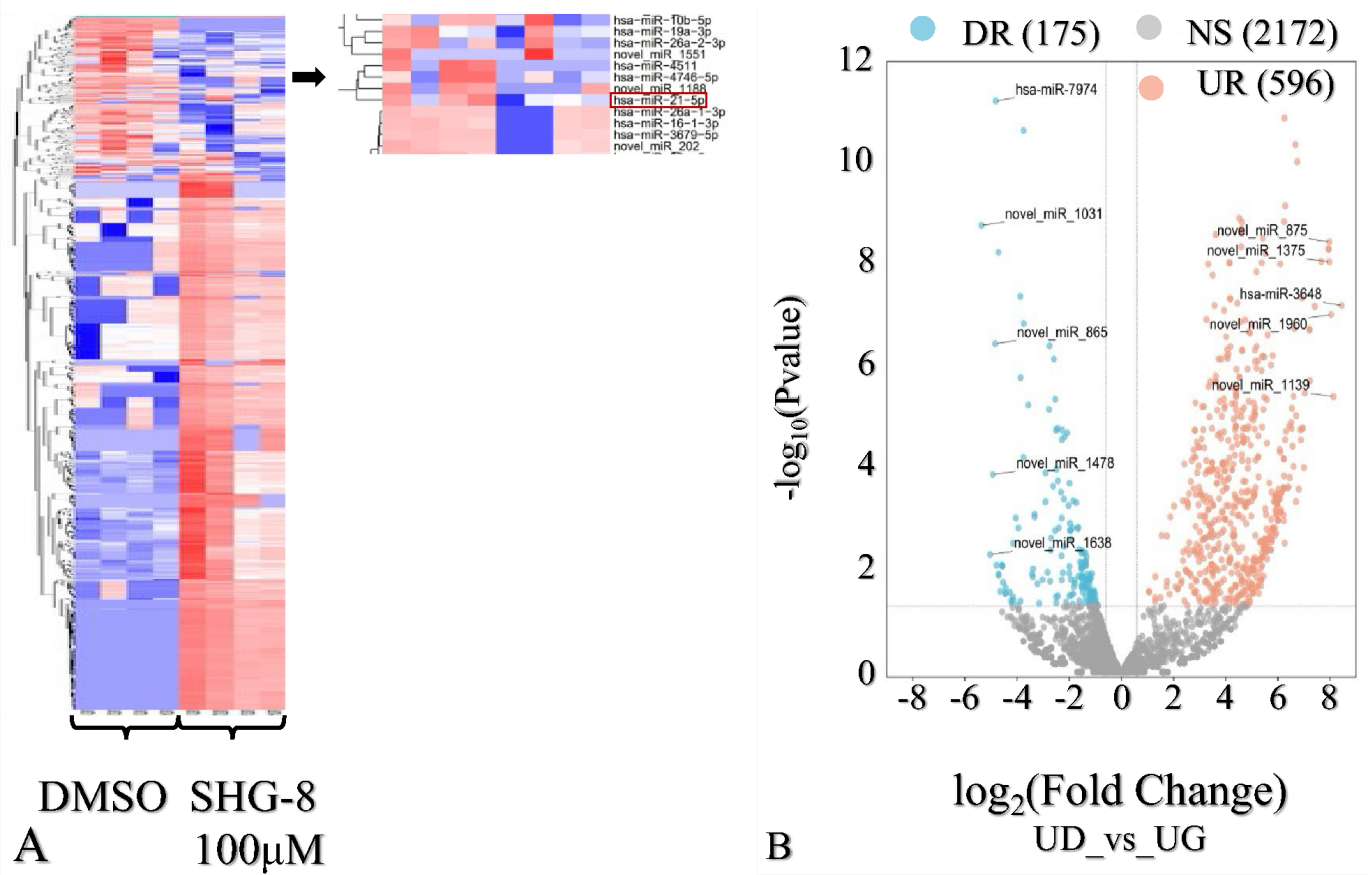
Comprehensive sRNA-seq analysis of SHG-8 treated samples. (A) Heatmap of deregulated miRNAs (groups: light blue, DMSO; light red, SHG-8 treated samples). (B) Volcano plot of miRNA expression revealed 771 deregulated miRNAs; 175 downregulated and 596 upregulated in the presence of SHG-8 (legend: NS, not significant; DR, downregulated; UR, upregulated).

sRNA-seq comprehensive miRNA expression and gene pathway analysis revealed that 771 miRNAs were either significantly upregulated or downregulated (Figure 7A). Of these, 175 miRNAs were downregulated, and 596 were upregulated in the SHG-8 treated conditions compared to the control samples (*q* value < 0.05 and |log2 fold change| > 1). The most downregulated miRNA was found to be miR-7974, which was associated with targeting 416 genes, while miR-3648 was the most upregulated miRNA, which targeted 212 genes (Figure 7B).

**Figure 8:**
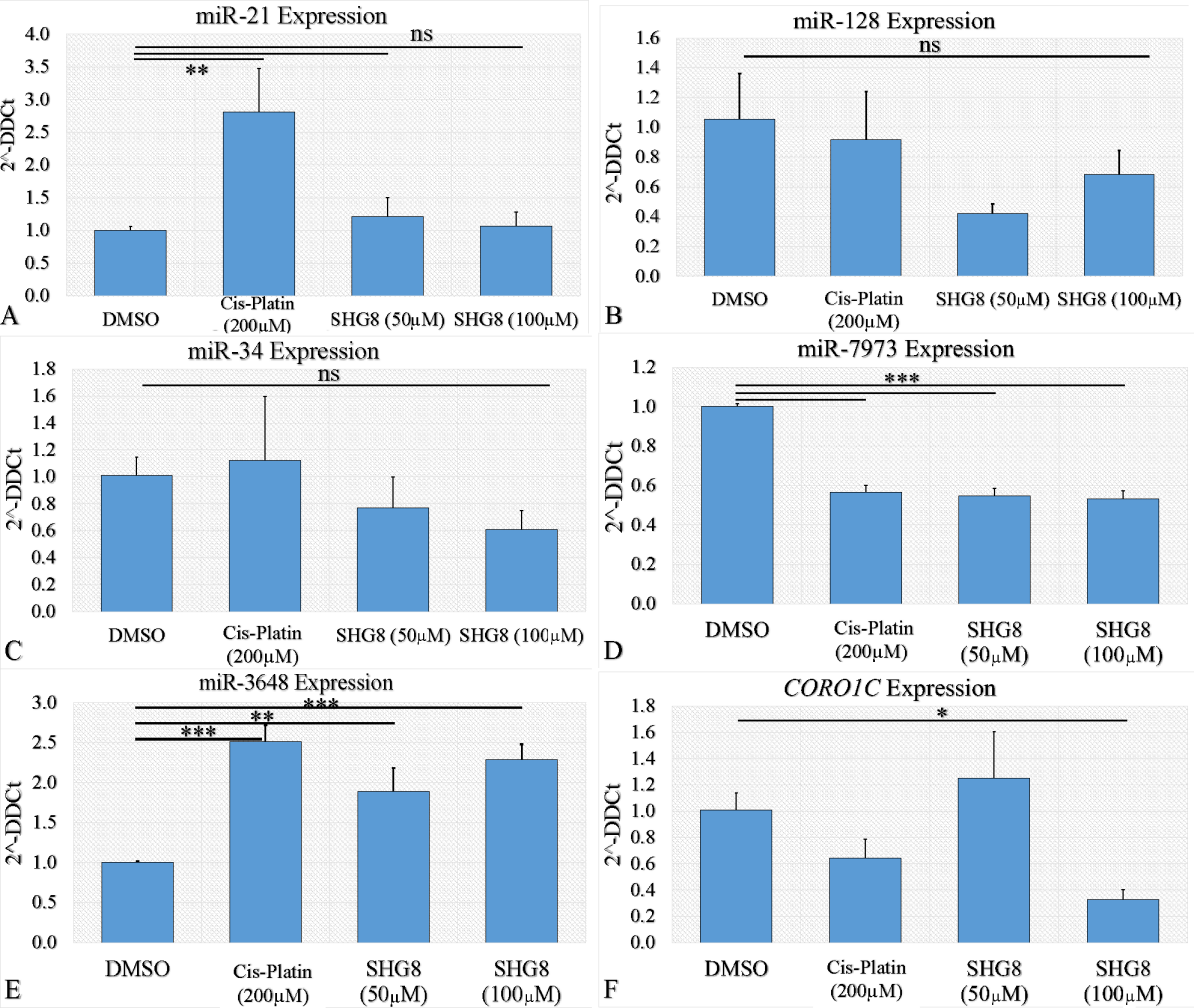
Relative RT-qPCR expression post exposure to SHG-8. Relative expression of miR-21 (A), miR-128a (B), miR-34a (C), miR-7973 (D), miR-3648 (E), and *CORO1C* (F) when treated with SHG-8 (*q* value < 0.05 and |log2 fold change| > 1; legend: ns, not significant, *p < 0.05, **p < 0.01, ***p < 0.001, ****p < 0.0001).

sRNA-seq miRNA analysis demonstrated that the commonly deregulated GB-specific miRNAs, miR-21-5p and miR-128a-3p were significantly downregulated. RT-qPCR results for these miRNAs did not reach a significant 2-fold change in expression (Figure 8A and B; *p* < 0.04 and *p* < 0.01, respectively). MiR-34a-5p (Figure 8C) showed no significant expression change in the sRNA-seq data and via RT-qPCR. RT-qPCR performed on miR-7974 (Figure 8D) and miR-3648 (Figure 8E) showed significant downregulation and upregulation, respectively. Furthermore, several genes involved in the Wnt/β-catenin pathway, such as *CORO2A*, *APC2*, and *WNT7A*, were direct targets of the most upregulated or downregulated miRNAs (hsa-miR-3648 and hsa-miR-7974, respectively). Thus, the differential expression of the *CORO1C*, a potential oncogene and a player in this pathway, was assessed via RT-qPCR and found to be significantly downregulated in the 100 μM SHG-8 condition with a 2-fold change in expression (*p* < 0.05; Figure 8F).

MiRNAs have emerged as promising therapeutic targets in GB treatment. Their small size and stability are advantageous over targeting protein-coding genes, making them attractive candidates for therapeutic interventions.^27^ Nevertheless miRNA-based therapies are not yet applicable due to their diverse binding abilities.

Thus, a response to a given drug may differ due to the multiple targets a single miRNA can regulate. To elucidate the action of SHG-8 upon the miRNA regulation within U87MG cells, sRNA-seq was conducted and revealed a plethora of deregulated miRNAs. The observed shift of miRNA expression profiles suggested that SHG-8 exerted prominent effects upon their regulation, which was further supported by the performed KEGG pathway analysis, which revealed that a sufficient number of miRNAs controlling genes were affected by the presence of SHG-8. The most downregulated miRNA was miR-7974, while the most upregulated miRNA was miR-3648, which were also confirmed by RT-qPCR. In support of these findings, Wang et al. also observed downregulation of miR-7974 within GB tissue samples.^28^ Administration of an miR-7974 mimic within gastric cancer influenced tumor cell proliferation, confirming its potential oncogenic properties.^29^ Thus, it can be hypothesized that miR-7974 could also act as an oncomiR within GB cells. The biological actions of miR-7974 within GB cells are yet to be elucidated, even though suggestions for its implication in the glycerophospholipid metabolism within tumor spheroids do exist.^30^

Regarding miR-3648, it has been associated with the inactivation of the Wnt/β-catenin signaling pathway in gastric cancer.^31^ Tang et al. observed that miR-3648 acted as a tumor-suppressive miRNA via a negative miR-3648/FRAT1-FRAT2/c-Myc feedback loop. Even though the exact mechanism of action of miR-3648 within GB is not fully understood, its strong association with the Wnt/β-catenin signaling pathway suggested possible implications as a therapeutic target.^31^

Overall, given that the expression changes of miR-3648 and miR-7974 are prominent and confirmed by both sRNA-seq and RT-qPCR, they are more likely to be considered as the main contributors of SHG-8-mediated cytotoxicity. However, this remains to be elucidated.

The effects of SHG-8 upon three miRNAs that are highly deregulated and involved in the tumorigenesis of GB, including miR-21, miR-128a, and miR-34a, were also investigated. miR-21 was found upregulated in gliomas, contributing to enhanced tumor cell survival and invasiveness, alongside its potential to induce resistance to various treatments, including chemotherapy and radiotherapy.^32^ Inhibition of miR-21-5p activity and suppression of *SOX2* resulted in reduced levels of β-catenin, leading to decreased invasiveness and migration. Consequently, the downregulation of miR-21-5p directly influenced the expression of β-catenin. In line with these findings, our sRNA-seq analysis showed that miR-21 was significantly downregulated by SHG-8, suggesting that reduced migratory and clonogenic abilities of glioma cells might be caused by the inhibition of miR-21. However, subsequent RT-qPCR analysis demonstrated unchanged miR-21 levels between the control and treatment conditions. This could be because real-time PCR is a more sensitive approach used in expression analysis, whereas sRNA-seq captures information on transcript isoforms and alternative splicing events.^33^ Additionally, cisplatin did not exhibit inhibitory effects upon miR-21. However, cisplatin has the potential to inhibit cell growth and proliferation via other mechanisms that are compensating for this. Furthermore, RT-qPCR analysis did not reveal significant changes in the expression of miR-34a and miR-128a. sRNA-seq analysis also demonstrated that the expression of miR-34a remained unchanged, while miR-128a was observed to be downregulated by the drug. miR-128a has shown downregulation in various malignancies, such as GB and bladder cancer.^34^

Reinstating the proper levels of miR-128a specific to the brain has demonstrated tumor-suppressive effects. This occurs through its interaction with the *E3F3a* and *BMI1* genes, resulting in decreased proliferation and invasiveness.^35^ Nevertheless, the SHG-8 influenced the downregulation of miR-128a, suggesting that the drug could not restore adequate levels of the miRNA, which could further suppress tumor proliferation and migration. On the contrary, miR-34a expression levels were not affected by the presence of SHG-8. miR-34a belongs to a family of tumor suppressor miRNAs, and its expression is significantly reduced in GB.^36^ miR-34 is known to be transactivated and controlled by the tumor suppressor protein P53. Moreover, miR-34a expression is negatively correlated with the expression of c-Met in GB cells, inhibiting GB tumor growth *in vivo* and induction of cell death.^26^ Thus, the unaffected expression of miR-34a is a limiting factor for SHG-8 due to its inability to enhance its expression and lead to desired tumor suppressive mechanisms. It is hard to elucidate the exact mechanisms underlying SHG-8 upon the discussed miRNA candidates; however potential downregulation or inhibition of miR-21 might serve beneficial for the suppression of tumor growth and metastasis.

KEGG pathway analysis of deregulated genes (DEG) revealed that they were primarily associated with axon guidance and microRNAs in cancer (*q* value < 0.05; Supplementary Figure 3A). Consecutive biological pathway analysis of DEG showed that the biological processes affected by SHG-8 were primarily associated with neuron development, homophilic cell adhesion via plasma membrane adhesion molecules, negative regulation of intracellular signal transduction, negative regulation of the Wnt/β-catenin pathway, and negative regulation of the neuron apoptotic process (*q* value < 0.01; Supplementary Figure 3B).

miR-21 and miR-3648 were both associated with the inactivation of the Wnt/β-catenin signaling pathway. The Wnt/β-catenin pathway plays a crucial role in controlling cancer progression. It is transduced through frizzled receptors and LRP5/LRP6 coreceptors, leading to a β-catenin signaling cascade. Some of the negative regulators influencing this pathway involve *APC*, *AXIN1*, *AXIN2*, and *PPARG*.^37^ Our sRNA-seq analysis revealed that multiple oncogenes, including *IDH2*, *FGFR3*, *IGFBP3*, *MADD*, *NF2*, *CORO2A*, *PDCD1/2*, and *GFAP* were negatively influenced by SHG-8 supporting its tumor suppressive properties (Supplementary Figure 3B). For instance, the *GFAP* gene encodes the production of glial fibrillary acidic protein, which belongs to the intermediate filament protein family. Intermediate filaments play a crucial role in regulating the shape, movement, and function of glial cells.^38^ Thus, the negative SHG-8 regulation exerted upon *GFAP* could lead to a disturbed glial balance and enhanced permeability within the blood–-brain barrier (BBB), allowing the passage of SHG-8 molecules and their direct targeting of GB. Furthermore, knocking out *MADD* (MAPK-activating death domain) within anaplastic thyroid cancer resulted in a notable decrease in cellular migration, invasion potential, and clonogenic capacity *in vitro*.^39^ Thus, SHG-8 regulated silencing of *MADD* might lead to antimigratory and anti-invasive effects within GB, which could be accompanied by the inhibition of epithelial-mesenchymal transition (EMT) and Wnt-signaling pathways. The scientific evidence regarding both *GFAP* and *MADD* correlated with the RNA-seq findings related to the biological processes influenced by SHG-8, which included the effect on neuron development, negative regulation of intracellular signal transduction, negative regulation of the Wnt-signaling pathway, and negative regulation of the neuron apoptotic process. To strengthen the hypothesis that SHG-8 negatively regulated the Wnt-pathway, an *in vitro* RT-qPCR of *CORO1C* revealed that the gene was significantly downregulated in the presence of the drug. *CORO1C* acts as an F-actin turnover effector influencing neurite overgrowth and migration of brain tumor cells.^40^ An elevated expression of *CORO1C* in metastatic malignancies, such as GB and colorectal cancer, was previously documented, indicating the potential clinical relevance of this gene as a biomarker associated with an unfavorable prognosis.^41^ The negative effects of SHG-8 upon this oncogene supported the findings that SHG-8 exerted negative regulation upon *de novo* neurone overgrowth and the Wnt pathway, thus limiting the proliferative and migratory abilities of U87MG GB cells. Furthermore, the high affinity binding between the CORO1C protein receptor and the SHG-8 ligand demonstrated in our molecular docking analysis suggested that GB cell inhibition might be partially through targeting CORO1C (Supplementary Figure 4, Supplementary Table 1).

### 3.7 SHG-8 Possessed Sustainable Bioavailability

The nine predicted metabolites (SHG-8-1 to SHG-8-9, Supplementary Figure 2) are based on one reduction of the parent compound SHG-8 by carbonyl reductase as well as a number of hydroxylations and dealkylations predicted to be carried out by cytochrome P450 1A2 (CYP1A2). A selected number of absorption, distribution, and toxicity properties of SHG-8 and metabolites predicted with admetSAR 2.0 are shown in Table 1. For comparison, the over-the-counter painkillers aspirin and ibuprofen were included in the prediction. From Table 1, SHG-8 has predicted absorption and distribution features comparable with aspirin and ibuprofen, which suggests that SHG-8 is sufficiently absorbed and distributed in the body. In particular, SHG-8 was predicted to pass the blood–brain barrier. SHG-8 and all metabolites did fall into the acute oral toxicity class III (medium), while class IV is the lowest toxicity class. With regards to various toxicity predictions, SHG-8 and metabolites were comparable to ibuprofen, except for inhibition of hERG, a gene that encodes for the alpha-subunit of a cardiac potassium channel, inhibition of which could potentially lead to heart problems. SHG-8 was not associated with carcinogenicity and nephrotoxicity. Furthermore, a number of metabolites were predicted positive for the micronucleus test, indicating a potential for genotoxicity. SHG-8 and metabolites were not predicted to be mutagenic or carcinogenic in the Ames test.

**Table 1:** Prediction of Absorption, Distribution, and Toxicity Properties of SHG-8 and Metabolites Together with Aspirin and Ibuprofen Predictions^a^

	compound										
feature^1^	SHG-8	SHG-8-1	SHG-8-2	SHG-8-3	SHG-8-4	SHG-8-5	SHG-8-6	SHG-8-7	SHG-8-8	aspirin	ibuprofen
Caco-2 permeability	+	+	–	+	–	+	–	+	+	–	+
human intestinal absorption	+	+	+	+	+	+	+	+	+	+	+
human oral bioavailability	+	+	+	+	+	+	–	+	+	+	+
blood–brain barrier	+	+	+	+	+	+	+	+	+	–	+
P-glycoprotein inhibitior	–	–	–	–	–	–	–	–	–	–	–
P-glycoprotein substrate	–	–	–	–	–	–	–	–	–	–	–
plasma protein binding^2^	0.791	0.725	0.972	0.918	0.972	0.821	0.649	0.897	0.760	0.583	0.751
Ames mutagenesis	–	–	–	–	–	–	–	–	–	–	–
acute oral toxicity^3^	III	III	III	III	III	III	III	III	III	II	III
carcinogenicity (binary)	–	–	–	–	–	–	–	–	–	–	–
hepatotoxicity	+	–	+	+	+	+	+	+	+	+	+
hERG inhibition	+	+	–	–	–	+	+	–	–	–	–
mitochondrial toxicity	–	+	+	+	+	+	–	+	–	+	+
micronucleus test	–	–	+	+	+	+	+	+	–	+	–
nephrotoxicity	–	–	–	+	–	–	–	–	–	+	–
reproductive toxicity	–	–	+	–	+	+	+	+	–	+	+
respiratory toxicity	–	+	+	–	+	–	–	–	–	+	+

a (a) A “+” symbol denotes a positive and a “–” symbol denotes a negative prediction or absence of a feature. (b) The numbers are fractions between 0 and 1. (c) The toxicity classes range from IV to I with class IV being the lowest possible toxicity class.

Cytotoxicity presents challenges in GB treatment. For instance, the selectivity of the BBB restricts the entry of many therapeutic agents into the brain, limiting their efficacy against GB cells, while potentially causing toxicity to healthy brain tissue.^42^ As predicted via the *in silico* analysis, SHG-8 can pass freely through the BBB, overcoming cytotoxicity-related challenges in GB treatment. The complex tumor microenvironment of GB and its rigid protection by the BBB can hinder the delivery and effectiveness of cytotoxic treatments.^43^ We demonstrated that SHG-8 has the capacity to negatively regulate genes involved in the maintenance of the BBB, leading to its weakening and thus exerting direct cytotoxic effects upon GB cells. The low oral toxicity possessed by SHG-8 metabolites could prove beneficial for patient comfort and better quality of life during therapy. Furthermore, a number of metabolites of SHG-8 were predicted as potential genotoxicity effectors, supporting the findings observed throughout the *in vitro* investigations. Genotoxicity is a valuable consideration in GB treatment as certain therapies, such as radiation and chemotherapeutic agents, including TMZ, can induce DNA damage and genomic instability.^44^ Nevertheless, it is essential to mention that this could lead to potential adverse effects on normal cells and contribute to the development of secondary malignancies.^45^

The present study of testing a new, potential anticancer agent within glioblastoma cell models could benefit from incorporating *in vivo* experimentations which will strengthen the validity of the results. In addition, animal models represent the tumor microenvironment better and thus would help to elucidate the specific drug efficacy and impact within model organism. Furthermore, the predicted genotoxicity of SHG-8 and its metabolites via the ADMET prediction highlights the need of further toxicity and efficacy evaluations before its clinical application. Our findings suggested that SHG-8 could act as a potential anticancer therapeutic and influences the miRNome of GB cells; however, sample size must be considered as a potential limitation.

In summary, the environmentally sustainable and economical synthesis of SHG-8 and its biological inhibitory effects upon GB cell viability, migration, and proliferation suggested that the compound might be a beneficial therapeutic agent against GB. SHG-8 demonstrated genotoxic properties via the release of ROS and promoting cells toward apoptotic stages. Inhibition of Wnt/β-catenin pathway-associated oncogenes, supported via the significant downregulation of miR-21 and *CORO1C*, might serve as a guiding point to understanding the mechanisms of action of the drug.

## Data Availability

The data presented in this research paper have been uploaded at Mendeley. Release date: 02/12/2023 (https://data.mendeley.com/datasets/v5mcf8hpsz/1).

## Supporting Information Available

Additional details on the methodology; supplementary figures on SHG-8 synthesis, metabolites of SHG-8, KEGG pathway and biological pathway bioinformatics analyses of sRNA-sequencing, and molecular docking of SHG-8; supplementary tables of molecular docking analysis and quantification of apoptosis (PDF)

## Abbreviations

GB: glioblastoma
miRNA: microRNA
RT-qPCR: real-time polymerase chain reaction
ROS: reactive oxygen species
β-AC: β-amino carbonyl
sRNA-seq: small RNA-sequencing
TMZ: temozolomide
TME: tumor microenvironment
SAFSNS: sulfonic acid-functionalized silica nanospheres
SHG-8: β3-(4-bromophenyl)-1-phenyl-3-(phenylamino)propan-1-one
MTT: 3-(4,5-dimethylthiazol-2-yl)-2,5-diphenyltetrazolium bromide
DMSO: dimethyl sulfoxide
GO: gene ontology
KEGG: Kyoto Encyclopaedia of Genes and Genomes
