# Supplementary material for: MicroRNA-Dependent Mechanisms Underlying the Function of a β-Amino Carbonyl Compound in Glioblastoma Cells
Source: ACS Omega. 2024 Jul 15;9(29):31789–802. doi: 10.1021/acsomega.4c02991 (PMC11270567; doi:10.1021/acsomega.4c02991)
Supplement: Supplementary file 1 — ao4c02991_si_001.pdf [file ao4c02991_si_001.pdf]

MICRORNA-DEPENDENT MECHANISMS UNDERLYING THE FUNCTION OF A β-AMINO CARBONYL COMPOUND IN GLIOBLASTOMA CELLS.

Denis Mustafov[1,2], Shoib S. Siddiqui[1], Andreas Kukol[1], George I. Lambrou[3,4], Shagufta[5], Irshad Ahmad[5], Maria Braoudaki[1,4,*]

[1]School of Life and Medical Sciences, University of Hertfordshire, Hatfield, AL10 9AB, UK
[2]College of Health, Medicine and Life Sciences, Brunel University London, Uxbridge UB8 3PH, UK.
[3]Choremeio Research Laboratory, First Department of Pediatrics, School of Medicine, National and Kapodistrian University of Athens, Athens, Greece, Thivon & Levadeias 8, 11527, Goudi, Athens, Greece
[4]University Research Institute of Maternal and Child Health & Precision Medicine, National and Kapodistrian University of Athens, Thivon & Levadeias 8, 11527 Athens, Greece
[5]Department of Biotechnology, School of Arts and Sciences, American University of Ras Al Khaimah, Ras Al Khaimah, United Arab Emirates (UAE).

Correspondence to:
*Prof. Dr Maria Braoudaki, Reader in Molecular Medicine
School of Life and Medical Sciences,
University of Hertfordshire,
College Lane Campus,
Hatfield AL10 9AB, United Kingdom (UK)
Email: m.braoudaki@herts.ac.uk

SUPPLEMENTARY METHODOLOGY

SM1. Synthesis of SHG-8

The sustainable sulfonic acid-functionalized silica nanospheres (SAFSNS) nano-catalyst was prepared and characterized according to Ahmad *et al*. (2021) (1). The desired compound SHG-8 was synthesized by the SAFSNS (0.04g) catalysed Mannich reaction of acetophenone (1.1mmol), 4-Bromo benzaldehyde (1mmol), and aniline (1mmol) in one pot synthesis by our reported procedure (2). The reaction mixture in ethanol at ambient temperature was stirred for four hours. The reaction progress was monitored by the TLC, and the solvent was evaporated to get a yellow solid product on completion of the reaction. The catalyst SAFSNS was separated from the solution of crude product in dichloromethane at 35°C by filtration. Subsequently, the reaction product was recrystallized in ethanol solvent as a light yellow solid and well characterised by the IR, ^{1}H-NMR, ^{13}C-NMR, and HRMS techniques.

SM2. MTT cell viability assay

Cell viability of U87MG and U251MG GB cells was performed as previously described by Vazhappilly el al. (2021). GB cells were seeded in 96-well plates at a cell density of 1.5×10^{4} per well and subsequently treated with increasing SHG-8 concentrations (diluted in DMSO, Dubai, UAE) of 20μM, 40μM, 60μM, 80μM, and 100μM for U87MG cells and 50μM, 100μM, 150μM, and 200μM for U251MG cells. Dimethylsulfoxide (DMSO) (ThermofisherTM, CA, USA) was used as a negative control, whereas cis-platin (200μM) (Sigma-AldrichTM, Dorset, UK) was used as a positive control. Following a 24h incubation with the treatment conditions, 5mg/mL 3-(4,5-dimethylthiazol-2-yl)-2,5-diphenyltetrazolium bromide (MTT) dye (GibcoTM, Bleiswijk, NL) was added in each well and plates were incubated for 2h at 5% CO_2 and 37°C. Afterwards, the MTT was removed, and 100% isopropanol (Sigma-AldrichTM, Dorset, UK) was introduced into each well and left for 30min on a rocking platform to dissolve formed formazan crystals. The absorbance values were measured at 590nm using a CLARIOstar microplate reader (BMG Labtech, Aylesbury, UK).

SM3. Colony forming assay

Colony formation potential of U87MG and U251MG cells was assessed as described by Vazhappilly *et al.* (2021) (3). Cells at a density of 500 cells/well were seeded in triplicates on 12-well plates and incubated with SHG-8 (50μM and 100μM) or <1% DMSO (negative control) or 200μM cis-platin (positive control), for 24 hours in complete media. Drug-supplemented media was removed the next day, and the plates were further incubated for five days at 5% CO_2 and 37°C in a humidifying incubator. Formed colonies were fixed with 4% paraformaldehyde (PFA) (Sigma-AldrichTM, Dorset, UK) for 25min, followed by staining with 0.1% crystal violet (Sigma-AldrichTM, Dorset, UK). The stained colonies were washed with distilled water and left to air dry overnight. Colonies containing more than 30 cells were microscopically photographed and quantitatively assessed under a light microscope (Olympus Life Science Solutions, Stansted, UK).

SM4. Scratch assay

A wound healing assay was performed as previously described by Vazhappilly *et al.* (2021) (3). U87MG and U251MG cells were seeded in a 12-well plate at a cell density of 2.5x10^5 cells/well. Scratches were performed as straight lines across the wells with a 2µL pipette tip before the addition of the treatment conditions, and the wells were washed twice with PBS to remove any cell debris lifted while performing the scratches. U87MG cells were treated with <1% DMSO (negative control) or 200µM cis-platin (positive control), and two concentrations of SHG-8 (20µM and 40µM). U251MG cells were treated with <1% DMSO (negative control) or 200µM cis-platin (positive control) and two concentrations of SHG-8 (50µM and 100µM). Images of the scratch midlines were taken at 0h, 24h, and 48h using a light microscope. The migration ability of the cells was analysed via using the ImageJ software with the assistance of a wound healing plugin (4, 5). The percentage of the healed area of the scratch at 24 and 48 hours was normalized to t_0 (0 hours) for all treatments to facilitate cross-comparisons.

SM5. RNA isolation and TaqMan expression assays

RT-qPCR was performed as described by Braoudaki *et al.* (2016) (6). In brief, total RNA and miRNAs were extracted following the Trizol reagent (Ambion Life Technology, Aukland, New Zealand) protocol and mirVana isolation kit (ThermoFisher, Vilnius, Lithuania), respectively. The sample's quantity and quality were assessed using Nanodrop (Nanodrop ND1000 Spectrophotometer, (Marshall Scientific, Hampton, USA). cDNA synthesis reactions were performed by using a Thermal cycler (Eppendorf, Mastercycler nexus gradient, ThermofisherTM, CA, USA) by using High-Capacity cDNA Reverse Transcription Kit (Applied Biosystems ThermoFisher, Pleasanton, CA). RT-qPCR experiments were performed by using QuantStudio™ Real-Time PCR (Quant Studio 7 flex, Applied Biosystems, Massachusetts, USA). For mRNA profiling, the thermal cycler ran at 25°C for 10min, followed by 37°C for 120min, and 85°C for 5min, whereas the PCR cycles ran at 95°C for 20sec, followed by 40 cycles of 95°C for 1sec and 60°C for 20sec. For miRNA profiling, the thermal cycler ran at 16°C for 30min, followed by 42°C for 30min, and 85°C for 5min, whereas the PCR cycles ran at 95°C for 10min, followed by 40 cycles of 95°C for 15sec and 60°C for 1min. Expression analysis in different samples was performed by using specific primers for each gene and miRNAs. $2^{-\Delta\Delta Ct}$ values of fold expression were used to compare the relative differences between the SHG-8 treated samples and the DMSO control samples.

SM6. Library preparation for sRNA sequencing

A total of 1.5µg RNA per sample was used as input material for the RNA sample preparations. Sequencing libraries were generated using NEBNext®Ultra™ small RNA Sample Library Prep Kit for Illumina (NEB, USA) following the manufacturer's recommendations, and index codes were added to attribute sequences to each sample. Firstly, ligated the 3′ SR Adaptor was made via mixing 3′ SR Adaptor for Illumina, RNA and Nuclease-Free Water. The mixture system was incubated for 2min at 70°C in a preheated thermal cycler. The tube was transferred on ice. Then, 3′ Ligation Reaction Buffer (2X) and 3′ Ligation Enzyme Mix ligate the 3′ SR Adaptor were added, and the mixture was incubated for 1 hour at 25°C in a thermal cycler. To prevent

adaptor-dimer formation, the SR RT Primer hybridizes to the excess of 3′ SR Adaptor (that remains free after the 3′ ligation reaction) and transforms the single-stranded DNA adaptor into a double-stranded DNA molecule. Secondly, ligation of the 5′ SR Adaptor was performed, followed by reverse transcription synthetic chain reaction. Lastly, PCR amplification and Size Selection followed. PAGE gel was used for electrophoresis fragment screening purposes and rubber cutting recycling as the pieces get small RNA libraries. PCR products were purified (AMPure XP system), and library quality was assessed on the Agilent Bioanalyzer 2100 system.

SM7. Clustering and sequencing

The clustering of the index-coded samples was performed on a cBot Cluster Generation System using TruSeq PE Cluster Kit v4-cBot-HS (Illumia) according to the manufacturer's instructions. After cluster generation, the library preparations were sequenced on an Illumina Hiseq 2500 platform and paired-end reads were generated.

SM8. Data analysis

Raw data (raw reads) of fastq format were first processed through in-house perl scripts. In this step, clean data (clean reads) were obtained by removing reads containing adapter, ploy-N and low-quality reads from raw data, and reads were trimmed and cleaned by removing the sequences smaller than 18 nucleotides or longer than 30 nucleotides. At the same time, Q20, Q30, GC-content and sequence duplication level of the clean data were calculated. All the downstream analyses were based on clean data with high quality.

SM9. Comparative analysis

Utilisation of the Bowtie tools software, in particular The Clean Reads with Silva database, GtRNAdb database, Rfam database, and Repbase database sequence alignment were incorporated in filtering ribosomal RNA (rRNA), transfer RNA (tRNA), small nuclear RNA (snRNA), small nucleolar RNA (snoRNA) and other ncRNA and repeats. The remaining reads were used to detect known miRNA and new miRNA predicted by comparing with known miRNAs from miRBase. The Randfold tools were used for the prediction of new miRNA secondary structure.

SM10. Target gene functional annotation

Gene function was annotated based on the following databases: Nr (NCBI non-redundant protein sequences: https://www.ncbi.nlm.nih.gov/refseq/); Nt (NCBI non-redundant nucleotide sequences: https://www.ncbi.nlm.nih.gov/refseq/) Pfam (Protein family: http://pfam.xfam.org); KOG/COG (Clusters of Orthologous Groups of proteins: http://www.pdg.cnb.uam.es/cursos/Leon2002/pages/software/DatabasesListNAR2002/summary/7.html#:~:text=Database%20Description,orthologs%20(direct%20evolutionary%20counterparts)); Swiss-Prot (A manually annotated and reviewed protein sequence database: https://www.uniprot.org); KO (KEGG Ortholog database: https://www.genome.jp/kegg/ko.html); GO (Gene Ontology: https://geneontology.org).

SM11. Quantification of miRNA expression levels

miRNA expression levels were estimated for each sample: 1. sRNAs were mapped back onto the precursor sequence. 2. The read count for each miRNA was obtained from the mapping results.

SM12. Differential expression analysis

For the samples with biological replicates: Differential expression analysis of two conditions/groups was performed using the DESeq R package (1.10.1). DESeq provides statistical routines for determining differential expression in digital miRNA expression data using a model based on the negative binomial distribution. The resulting p values were adjusted using Benjamini and Hochberg's approach for controlling the false discovery rate. miRNA with an adjusted $p<0.05$ found by DESeq were assigned as differentially expressed. For the samples without biological replicates: Prior to differential gene expression analysis, for each sequenced library, differential expression analysis of two samples was performed using the IDEG6. The p value was adjusted using q value (7). Q value<0.005 & |log2 (foldchange) |≥1 was set as the threshold for significantly differential expression.

SM13. GO enrichment analysis and KEGG pathway enrichment analysis

Gene Ontology (GO) enrichment analysis of the differentially expressed genes (DEGs) was implemented by the GOseq R packages based on Wallenius non-central hyper-geometric distribution. KEGG is a database resource for understanding high-level functions and utilities of the biological system, such as the cell, the organism, and the ecosystem, from molecular-level information, especially large-scale molecular datasets generated by genome sequencing and other high-throughput experimental technologies (http://www.genome.jp/kegg/) (8). We used KOBAS software to test the statistical enrichment of differential expression genes in KEGG pathways (9).

SUPPLEMENTARY FIGURES AND TABLES

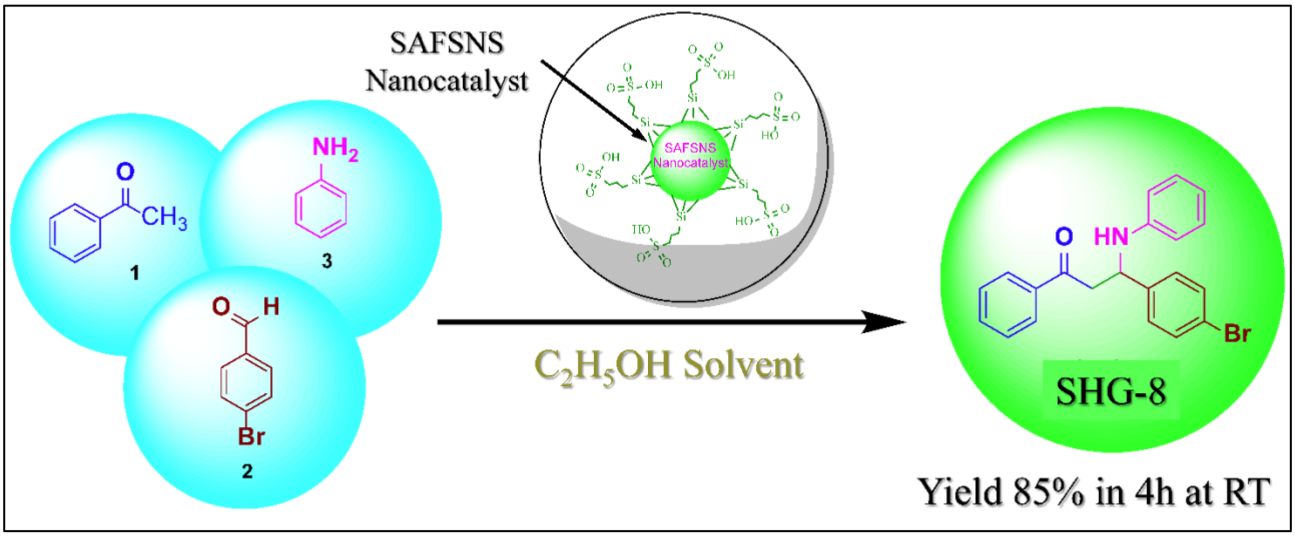

Supplementary Figure 1. Three-component reaction of acetophenone, 4-bromo benzaldehyde, and aniline catalysed by SAFSNS to synthesise the target compound SHG-8. The synthesized SAFSNS catalyst was applied efficiently for the synthesis of a target molecule, 3-(4-bromophenyl)-1-phenyl-3-(phenylamino) propane-1-one (SHG-8) through the Mannich reaction. An efficient, simple, and green catalytic process was applied for the one-pot condensation of aromatic ketone (1), aromatic aldehyde (2), and aromatic amine (3) using an environmentally benign solid acid catalyst (**Abbreviations**: **RT**: Room Temperature).

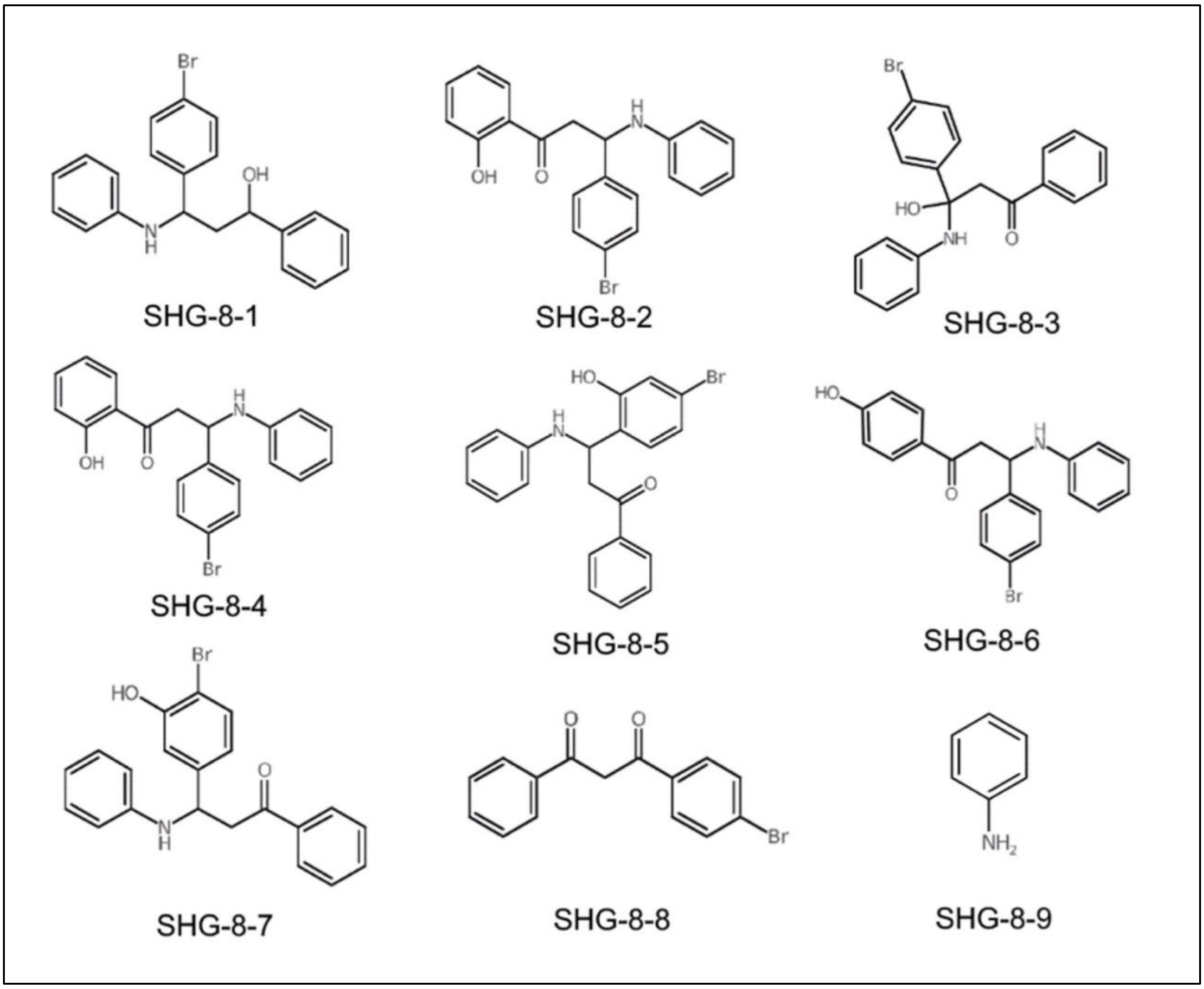

Supplementary Figure 2. Metabolites of SHG-8 predicted by Biotransformer 3.1.

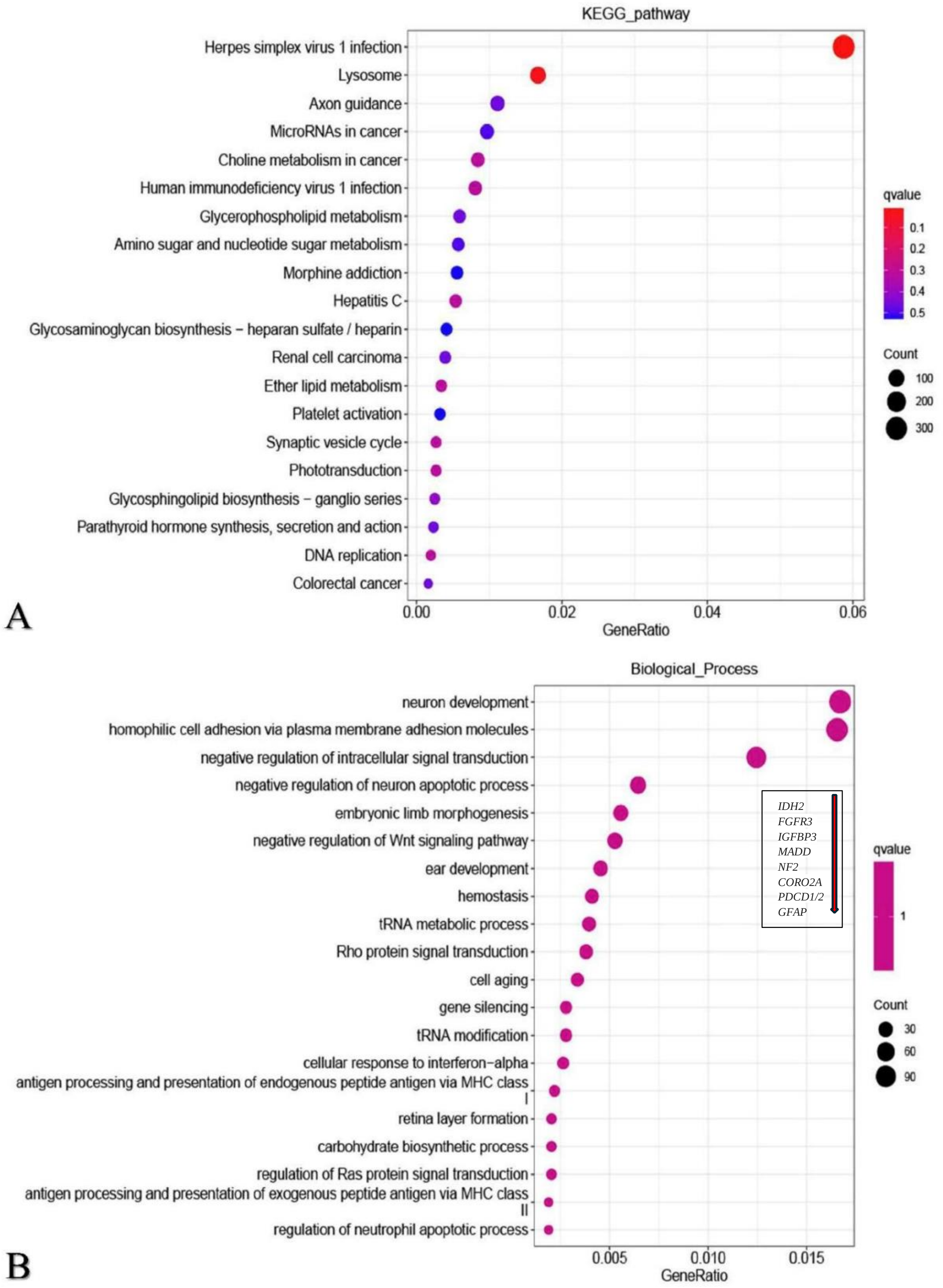

Supplementary Figure 3. Comprehensive pathway sRNA-seq analysis of SHG-8 treated samples. KEGG pathway DEG analysis revealed that genes were primarily associated with relevant pathways e.g "microRNAs in cancer" (**A**). Biological pathway analysis of DEG showed that biological processes were primarily associated with neuron development, negative regulation of intracellular signal transduction, negative regulation of Wnt-signaling pathway, and negative regulation of neuron apoptotic process. *IDH2, FGFR3, IGFBP3, MADD, NF2, CORO2A, PDCD1/2, GFAP* were downregulated by SHG-8 (**B**).

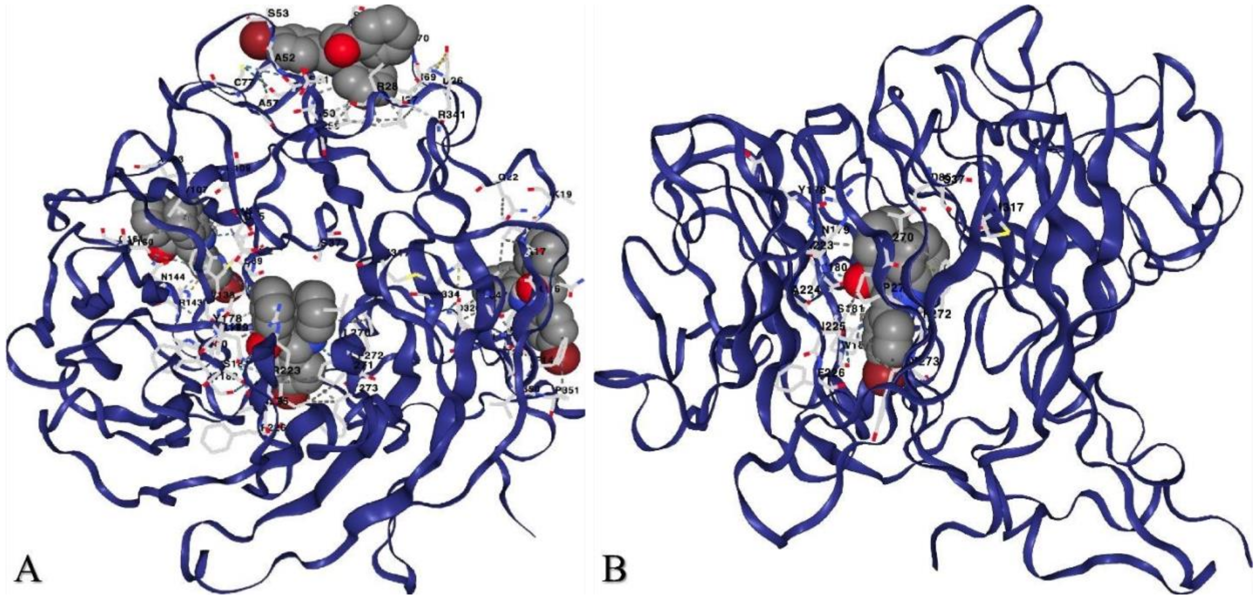

Supplementary Figure 4. Molecular docking analysis between CORO1C and SHG-8. The cartoon illustrated the five binding sites between CORO1C and SGH-8 in a spacefill model **(A)**. The cartoon visualised the strongest binding force between SHG-8 and CORO1C at position C1 defined by the lowest binding energy of -8.1 (Vina score) **(B)**.

Supplementary Table 1. Molecular docking sites and binding affinities between the large molecule receptor protein CORO1C and the small molecule ligand SHG-8.

CurPocket ID	Vina score	Cavity volume (Å3)	Center (x, y, z)	Docking size (x, y, z)
**C1**	-8.1	3858	54, 18, 9	22, 35, 34
**C5**	-7.6	132	49, 21, -23	22, 22, 22
**C2**	-7.2	196	74, 2, -5	22, 22, 22
**C3**	-6.5	178	39, 4, -2	22, 22, 22
**C4**	-6.3	139	70, 9, -10	22, 22, 22

Supplementary Table 2. Quantification of apoptotic bodies of U251MG and U87MG cells following 24h SHG-8 exposure.

	DMSO	Cis-platin 200µM	SHG-8 50µM	SHG-8 100µM
**U87MG Apoptosis**	0%	48.66% ± 5.7%	0%	98.33% ± 0.9%
**U87MG Necrosis**	4% ± 1.6%	34% ± 4.8%	9% ± 3.2%	1.33 ± 0.4%
**U251MG Apoptosis**	0%	41% ± 2.1%	91.33% ± 3.3%	78.66 ± 4.1%
**U251MG Necrosis**	6.33% ± 1.2%	57.66% ± 5.3%	8.66% ± 2.4%	18.11% ± 6.1%

REFERENCES

1. Ahmad I, Shagufta, Dhar R, Hisaindee S, Hasan K. An Environmentally Benign Solid Acid Nanocatalyst for the Green Synthesis of Carboxylic Acid Ester. ChemistrySelect. 2021;6(36):9645-52. doi.https://doi.org/10.1002/slct.202102230.

2. Ahmad MS, Braoudaki M, Patel H, Ahmad I, Shagufta, Siddiqui SS. Novel Siglec-15-Sia axis inhibitor leads to colorectal cancer cell death by targeting miR-6715b-3p and oncogenes. Frontiers in immunology. 2023;14:1254911. doi.10.3389/fimmu.2023.1254911.

3. Vazhappilly CG, Hodeify R, Siddiqui SS, Laham AJ, Menon V, El-Awady R, et al. Natural compound catechol induces DNA damage, apoptosis, and G1 cell cycle arrest in breast cancer cells. Phytotherapy research : PTR. 2021;35(4):2185-99. doi.10.1002/ptr.6970.

4. Schneider CA, Rasband WS, Eliceiri KW. NIH Image to ImageJ: 25 years of image analysis. Nature methods. 2012;9(7):671-5. doi.10.1038/nmeth.2089.

5. Suarez-Arnedo A, Torres Figueroa F, Clavijo C, Arbeláez P, Cruz JC, Muñoz-Camargo C. An image J plugin for the high throughput image analysis of in vitro scratch wound healing assays. PloS one. 2020;15(7):e0232565. doi.10.1371/journal.pone.0232565.

6. Braoudaki M, Lambrou GI, Papadodima SA, Stefanaki K, Prodromou N, Kanavakis E. MicroRNA expression profiles in pediatric dysembryoplastic neuroepithelial tumors. Medical oncology (Northwood, London, England). 2016;33(1):5. doi.10.1007/s12032-015-0719-3.

7. Storey JD, Tibshirani R. Statistical significance for genomewide studies. Proceedings of the National Academy of Sciences of the United States of America. 2003;100(16):9440-5. doi.10.1073/pnas.1530509100.

8. Kanehisa M, Araki M, Goto S, Hattori M, Hirakawa M, Itoh M, et al. KEGG for linking genomes to life and the environment. Nucleic acids research. 2008;36(Database issue):D480-4. doi.10.1093/nar/gkm882.

9. Mao X, Cai T, Olyarchuk JG, Wei L. Automated genome annotation and pathway identification using the KEGG Orthology (KO) as a controlled vocabulary. Bioinformatics (Oxford, England). 2005;21(19):3787-93. doi.10.1093/bioinformatics/bti430.
